# Estrogen alleviates post-traumatic osteoarthritis progression and decreases p-EGFR levels in female mouse cartilage

**DOI:** 10.1186/s12891-022-05608-y

**Published:** 2022-07-19

**Authors:** Zhihua Lu, Aihua Zhang, Jingcheng Wang, Kuijing Han, Han Gao

**Affiliations:** 1 Yangzhou Polytechnic College, Yangzhou, Jiangsu 225009 People’s Republic of China; 2 Department of Rehabilitation Medicine, Clinical Medical College of Yangzhou University, Northern Jiangsu People’s Hospital, Yangzhou, Jiangsu 225001 People’s Republic of China; 3 Department of Orthopedics, Clinical Medical College of Yangzhou University, Northern Jiangsu People’s Hospital, Yangzhou, Jiangsu 225001 People’s Republic of China; 4 Department of Doppler Ultrasonic, Clinical Medical College of Yangzhou University, Northern Jiangsu People’s Hospital, Yangzhou, Jiangsu 225001 People’s Republic of China

**Keywords:** Estrogen, Osteoarthritis, Cartilage, Epithelial growth factor receptor

## Abstract

**Objective:**

To investigate the effect of estrogen on the progression of post-traumatic osteoarthritis (PTOA) in mice and its possible mechanism.

**Methods:**

Twelve-week-old ICR mice were divided into Group A (female control group), group B (ovariectomized(OVX) group), group C (OVX group supplemented with estrogen), and group D (male group) by destabilization of the medial meniscus (DMM)or sham operation. Safranin O staining was performed at 8 weeks and 12 weeks after operation, and the degree of articular cartilage lesion was evaluated using Mankin score. Twelve weeks after the operation, tissue sections were stained to analyze the matrix metalloproteinase 13(MMP13), phosphorylated epidermal growth factor receptor (p-EGFR) expression and apoptosis of chondrocytes.

**Results:**

Decreased estrogen can significantly increase the weight of mice in female mice. The degree of cartilage damage in the knee joint on the DMM side of female mice was significantly severer than that on the Sham side. The DMM side also showed higher MMP13 expression and increased apoptotic chondrocytes. The degree of cartilage damage in the knee joint on the DMM side of female mice was significantly reduced after estrogen supplementation, and cartilage damage in the knee joint on the DMM side of female mice was less serious than that of male mice. As estrogen levels decreased, the severity of cartilage erosion in the knee joint on the DMM side was aggravated, and p-EGFR expression in the cartilage surface was also higher in female mice contrast to that in male mice. However, minimal changes in p-EGFR expression in the cartilage surface of bilateral knee joints of male mice were observe.

**Conclusion:**

Estrogen has a regulatory effect on PTOA and its inhibits the expression of p-EGFR in cartilage on the knee joint surface and has a protective effect on articular cartilage in female mice.

## Introduction

Osteoarthritis (OA) is a common whole joint disease affected l0 billion patients worldwide, and articular cartilage degeneration, osteophyte formation, subchondral sclerosis and synovitis are main pathological manifestations [1], among which cartilage erosion plays a central role in OA progression.

Articular cartilage is an important part of bone joints and mainly includes the calcified and uncalcified layers. The destruction of the cartilage during OA is mainly caused by the upregulation of MMPs. MMPs are key enzyme mediating the degradation of all types of collagen in the extracellular matrix [2]. Recent studies have confirmed [3] that MMP13 expression is a key target in the progression of OA lesions. High MMP13 expression has been observed in the articular chondrocytes of OA patients [4]. Similarly, overexpression of MMP13 in the articular cartilage of transgenic mice leads to the excessive degradation of chondrocytes in the extracellular matrix, especially their outer structure and accelerates the development of OA [5]. Therefore, increased MMP13 expression greatly accelerates OA progression.

Eestrogen may mediate the pathophysiological process of the onset of OA, inhibit the destruction of the articular cartilage, and delay the progression of OA. Although most studies have confirmed the protective effect of estrogen on the development of OA, the effect of estrogen on cartialge metabolism remains incompletely understood. Among 22 animal studies [6], only 11 reported the beneficial effects of estrogen on OA, and six even showed harmful effects. Martin et al. [7] believed that estrogen plays a dual role of as an anti-inflammatory and pro-inflammatory agents in the pathogenesis of OA. Klerk et al. [8] found insufficient evidence of a connection between estrogen and OA. In fact, scholars are only beginning to understand the effect of estrogen on joint tissues during the occurrence and development of OA; moreover, the specific mechanism of estrogen in OA is unclear.

In recent years, studies [9–11] demonstrated that EGFR signaling pathway plays an important role in the development, metabolism, and pathological changes of the articular cartilage. Many studies conclude that activation of the EGFR signaling pathway can accelerate the pathological changes of articular cartilage [12, 13]. However, the direct role of EGFR signaling pathway in OA and its pathological mechanisms have not been fully elucidated. Current studies on the EGFR signaling pathway focused on the related ligands (e.g., epidermal growth factor, transforming growth factor, epidermal regulator, bidirectional regulator) and the intracellular inhibitor mitogen-inducible gene 6 [9]. Studies have found that the EGFR signaling pathway may present gender-based effects in the process of PTOA in mice [12, 14]. Studies on lung adenocarcinoma have shown [15, 16] that the EGFR ligand is rapidly released to activate the EGFR and mitogen-activated protein kinase signaling pathways when lung cancer cells are stimulated with estrogen. Breast cancer studies [17] have revealed a close relationship between estrogen and the EGFR signaling pathway. Therefore, we hypothesized that an interaction mechanism exists between estrogen and the EGFR signaling pathway in articular cartilage. In this work, we performed DMM and OVX surgery on mice and explored the relationship between estrogen change, OA progression and cartilage erosion, trying to explore the effects of estrogen level on OA.

## Materials and methods

### Animals, surgery and treatment

48 healthy ICR female mice (age, 12 weeks old; weight, 34.38 ± 1.30 g) and 16 ICR male mice (age, 12 weeks old; weight, 42.25 ± 1.17 g) were purchased from the Comparative Medical Center of Yangzhou University and were housed in a temperature- and humidity-controlled environment (22 °C; 50% humidity) with a 12-h light/dark cycle.All experiments related to the use of animals were approved (approval no. YZPC 2,018,010) by the Institutional Animal Care and Use Committee of Yangzhou polytechnic college. The 48 female mice were randomly divided into groups A (female control group), B (OVX group), and C (OVX group supplemented with estrogen), each with 16 mice. All of the male mice were categorized as group D (male group). All mice were fasted for 6 h before surgery, weighed 1 h before surgery, and then anesthetized by an intraperitoneal injection of 6% pentobarbital sodium at a dose of 70-80 mg/kg. Surgeries were performed on all mice’s right knee for DMM and left knee for Sham. Mice of group B and C were ovariectomized and mice of group C were also implanted subcutaneously with pellets containing E2(17β-ESTRADIOL, cat. no.SE-121, 0.72 mg/90 day) according to manufacturer’s suggestion. These doses correspond to physiological levels of E2 in female mice. Briefly, for DMM, the joint capsule was opened and the medial meniscotibial ligament was cut to destabilize the meniscus without damaging other tissues. For sham, the joint capsule was opened to visualize medial meniscotibial ligament but without transaction. For OVX, varies were removed through a midline incision of the skin. The activity of the mice was observed daily and weighed every 4 weeks. 8 weeks, and 12 weeks later, they were killed by over anesthesia.. After euthanasia, knee joints were harvested for histology staining by Safranin O/Fast green to evaluate the change of the joint by Mankin score and quantification of the cartilage thickness, immunostaining of MMP13, p-EGFR and Tunnel staining to explore the mechanisms. This study is reported in accordance with ARRIVE guidelines.

### ELISA

Blood samples were taken from the hearts of the mice 8 and 12 weeks after surgery. Coagulated at room temperature 10–20 min, centrifugated 20 min at the speed of 2000–3000 r.p.m. to remove supernatant, If precipitation appeared, centrifugated again. To determine the concentrations of Estrogen in the serum, Estrogen BPA Environmental ELISA kits (abcam, cat. no. ab175820) were used according to the manufacturer's protocol.The absorbance was measured using a Multiskan MS 352 microplate reader (Thermo Fisher Scientific) at a wavelength of 450 nm. All the samples were thawed once and assayed in triplicate.

### Histology

The bilateral knee joints of the mice were collected, the surrounding soft tissue was thoroughly cleaned, and the joints were fixed with 4% paraformaldehyde at 4 °C for 48 h. Next, 10% EDTA was used for decalcification for 4 weeks, after which the knee joints were embedded in paraffin. A 6 μm-long continuous section was drawn along the medial bone cortex of each knee joint until the anterior cruciate ligament could be seen in the tissue section under a microscope. After slicing, two specimens were selected from every six specimens for safranin O staining (Beijing Solarbio Science & Technology Co., cat. no. G1371). The samples were stained, the tissue structure was observed under a light microscope, and about 7–9 specimens are selected from each joint for Mankin scores [18] analysis (cartilage structure [0 ~ 6], chondrocytes [0 ~ 3], and safranin O staining intensity [0 ~ 5]). The most sever specimen was picked up and analyzed by two independent observers. The Mankin score for each joint was the average score from the two observers. The tissues were classified according to Mankin scores as early OA (1 ~ 5), OA (5 ~ 9), and advanced OA (10 ~ 14).

According to the results of Mankin score, the section specimen with the most severe cartilage lesion was selected for each tissue, and the total cartilage thickness, uncalcified cartilage thickness and cartilage area were measured using ImageJ software (version 1.43, National Institutes of Health). The cartilage thickness was measured at five points exhibiting an average distribution of the articular cartilage layer of the tissue, and the average thickness was calculated and reported as the thickness of the articular cartilage. The average standard deviation of each group was compared.

### Immunohistochemistry and TUNEL

Histological analyses. Tissue samples were fixed in 4% paraformaldehyde buffered with phosphate-buffered saline (PBS, pH 7.4) at 4℃ for 1 day. Specimens were decalcified with 10% EDTA (pH 7.4) at 4℃ for 4 weeks, embedded in paraffin, and 6-µm thick sagittal sections were cut from specimens. Safranin O staining was performed according to standard protocols. For immunohistochemistry, sections were incubated with antibodies against MMP13 (1:200, abcam, cat. no. ab219620), p-EGFR (1:300, abcam, cat. no. ab40815). For immunofluorescence, a CSA II Biotin-Free Catalyzed Amplification System (Agilent Technologies) and Hoechst 33,258 (Agilent Technologies) counterstain were used.

TUNEL staining was performed with an In Situ Cell Death Detection Kit (Roche, Inc, cat. no. 11684817910)according to the manufacturer’s instructions. Histological analyses were performed at least three times using per group for confirmation of results. Images were visualized under a fluorescence microscope (Keyence, Osaka, BZ-X710). Counts of immunofluorescence-positive cells were calculated in five independent squares (100 × 100 µm) of articular cartilage harvested from the mouse medial tibial plateau, using BZ analyser software (Keyence). The same parameters were used for all acquisitions, and representative pictures are shown in the figures.

### Statistical Analyses

All data in each group were expressed as mean ± standard deviation (mean ± SD). Statistical analysis was carried out by SPSS18.0. One-way analysis of variance was used to compare the statistical differences among groups, and LSD test was used to compare the statistical differences among groups. T-test was used between independent samples. The experimental drawing was made by GraphPadPrism6 software. A p-value less than 0.05 was defined as statistically significant.

## Result

### Serum estrogen levels in mice

**Fig. 1:**
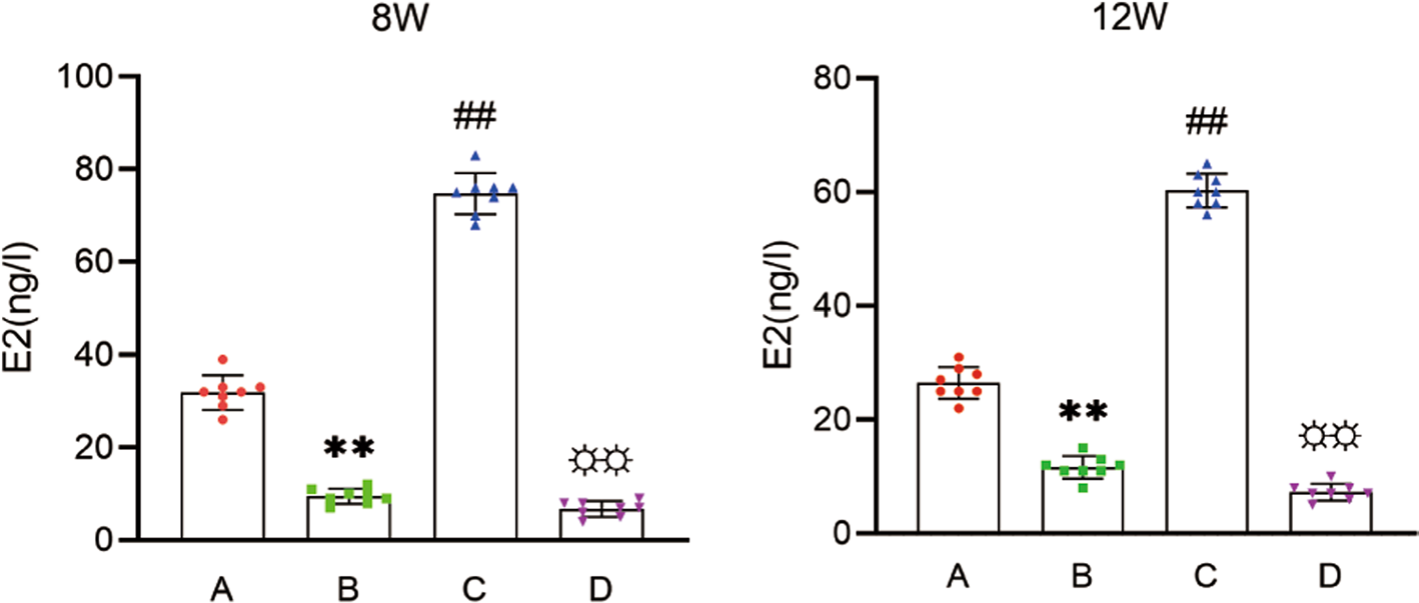
Serum estrogen levels of mice. (**A**). female control group; (**B**). ovariectomized group; (**C**). ovariectomized group supplemented with estrogen; and (**D**). male group; Group B compared with group A (* * *P* < 0.01)), group C compared with group B (# # *P* < 0.01)), and group D compared with group A (☼☼ *P* < 0.01)

We accurately measured serum estrogen levels in mice at 8 and 12 weeks after surgery by collecting blood from the heart. The serum estrogen level in group A was significantly higher than that in group D (Fig. 1). Compared with group A, the level of estrogen in group B decreased significantly following castration (Fig. 1). The level of serum estrogen in group C was significantly higher than that in group B (Fig. 1).

### Changes in the body weight of mice

**Fig. 2:**
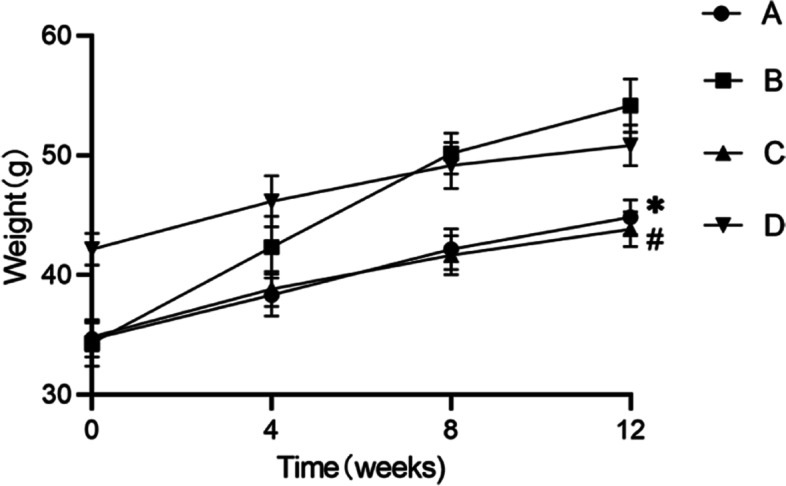
Changes in the body weight of mice. (**A**). female control group; (**B**). ovariectomized group; (**C**). ovariectomized group supplemented with estrogen; and (**D**). male group; Group A compared with groups B (* *P* < 0.01)). Group B group compared with group C (# *P* < 0.01)

The body weight of the mice was measured every 4 weeks after surgery to study the effect of estrogen on body weight. The weight of mice in each group increased to varying degrees over the 12-week study (Fig. 2). At the end of the experiment, the weight gain of mice in group B was the most obvious among the groups studied. Compared with that in group A, the weight of mice in group C increased by 13% (Fig. 2). The weight of mice in group C was 17% less than that in group B (Fig. 2). This result showed that a lack of estrogen promoted weight gain in mice, and that maintaining the physiological level of estrogen can inhibit the rapid growth gain body weight of mice to a certain extent.

### Pathological changes in the knee cartilage after medial meniscus instability in female mice

**Fig. 3:**
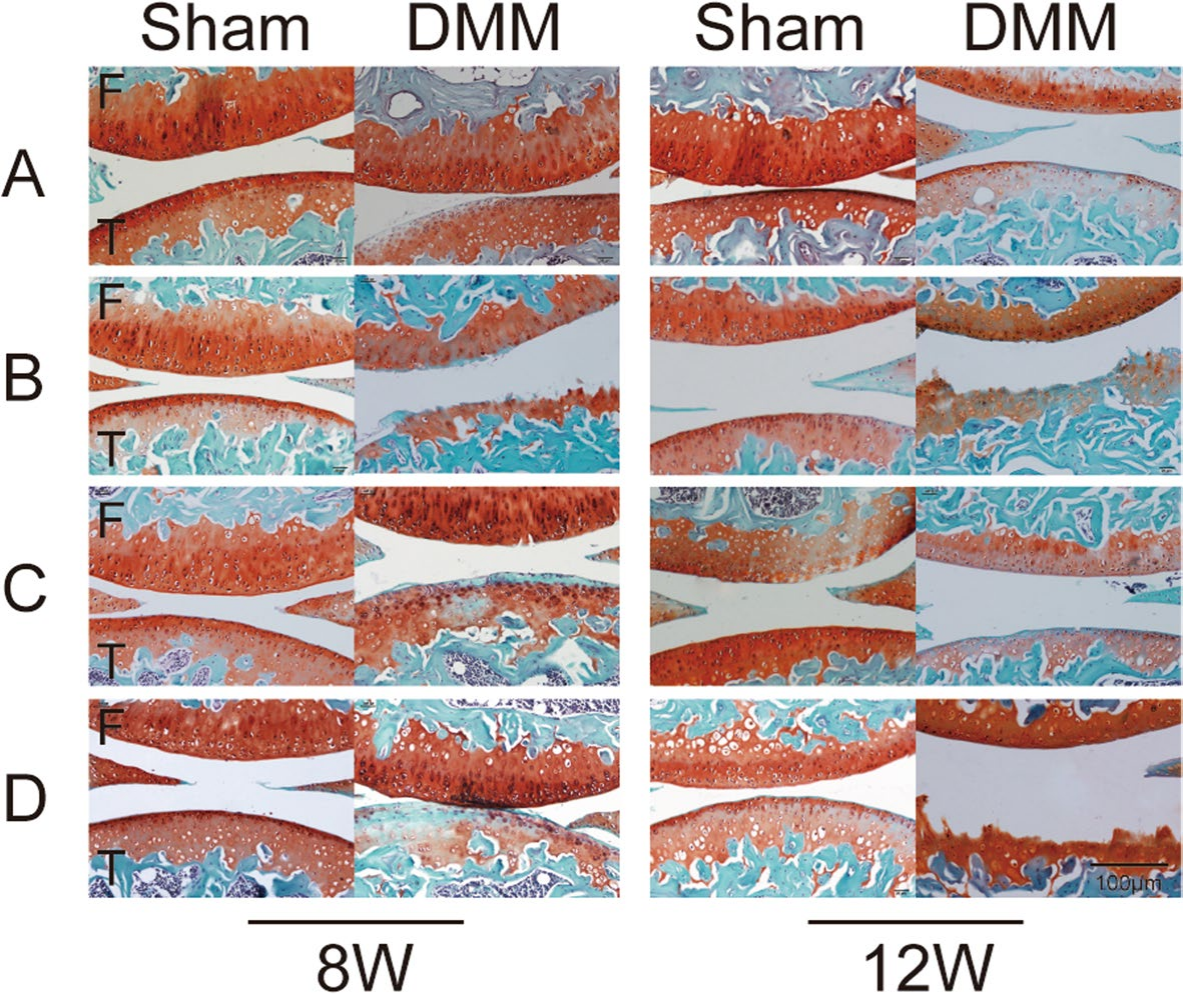
Safranin O staining of pathological sections of the knee joint of mice in groups. (Scale bar = 100 μm). (**A**). female control group; (**B**). ovariectomized group;( **C**). ovariectomized group supplemented with estrogen; and (**D**). male group; The blue area represents the subchondral bone, and the red area represents the articular cartilage.F. femour. T. tibia. Sham, sham operation; DMM, medial meniscus instability operation

**Fig. 4:**
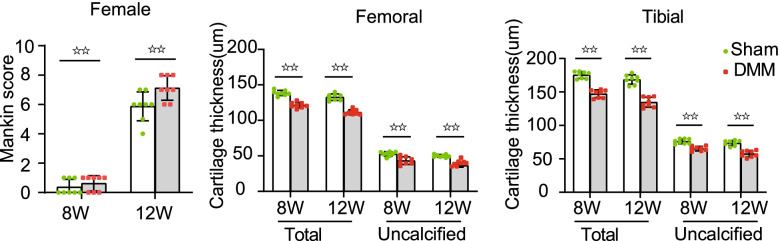
Mankin score and cartilage thickness of the bilateral knee joints of mice In group A (female control group). Sham, sham operation; DMM, medial meniscus instability operation; total, total cartilage; uncalcified, uncalcified cartilage; DMM was compared with Sham on both sides (☆☆ *P* < 0.01)

We evaluated the articular cartilage injury of female mice in group A to verify the modeling effect of DMM operation (Fig. 3, Fig. 4). Safranin O staining (macroscopic observation and Mankin score) and articular cartilage thickness were selected as evaluation indices for comparison. Articular cartilage injury on the DMM side was more severer than that on the Sham side in group A 8 and 12 weeks after operation A; Significant differences in Mankin score, articular cartilage and uncalcified cartilage thickness were also noted (Fig. 3, Fig. 4). Eight weeks after operation, moderate OA lesions were found in the DMM joints of group A; moderate-to-severe loss of safranin O staining, but no significant change in cartilage structure (5.87 ± 0.99points) were also noted. The thickness and area of articular and uncalcified cartilage decreased significantly (Fig. 3, Fig. 4). Twelve weeks after operation, pathological changes in the articular cartilage on the DMM side were further aggravated. Severe PTOA lesions were noted, and Mankin scores increased to 7.12 ± 0.83. The thickness and area of uncalcified and articular cartilage also decreased significantly. No obvious PTOA manifestation of the knee joint or damage to the articular cartilage was found on the Sham side. Although the data increased slightly, no statistical significance was determined at 12 weeks compared with that at 8 weeks (Fig. 4).

### Pathological changes in the knee cartilage induced by estrogen in mice

**Fig. 5:**
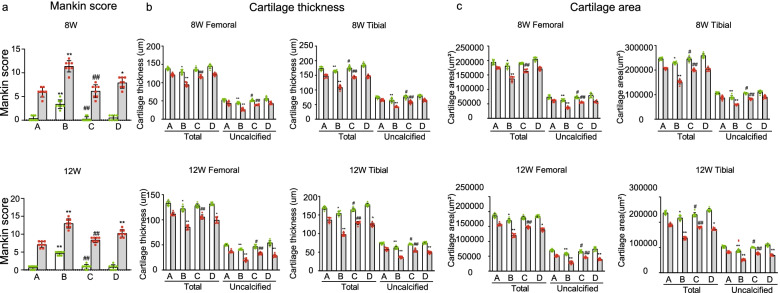
Comparative analysis of knee cartilage lesions in mice. (**A**). female control group; (**B**). ovariectomized group; (**C**). ovariectomized group supplemented with estrogen; and (**D**). male group; Sham, sham operation; DMM, medial meniscus instability operation; total, total cartilage; uncalcified, uncalcified cartilage; Ipsilateral comparison between groups B, D, and A (* *P* < 0.05,** *P* < 0.01). Ipsilateral comparison between groups C and B (# *P* < 0.05,## *P* < 0.01)

The cartilage injury of DMM knee joints was more severer in group D than in group A (Fig. 3, Fig. 5a-c). Mankin scores obtained 8 weeks after operation were compared with those of group A, and lesion differences increased 12 weeks after operation (Fig. 5a). Significant differences in the thickness and area of articular and uncalcified cartilage were observed between groups D and A 12 weeks after operation (Fig. 3, Fig. 5b and c).

At each time point investigated after surgery, the degree of cartilage injury in both sides of the knee joint in group B was more severe than that in group A (Fig. 3, Fig. 5a-c). Eight weeks after surgery, the DMM knee joint in group B showed severe PTOA lesions. Moreover, histology showed a decrease in safranin O staining, a large area defective uncalcified cartilage, and serious decreases in the area and thickness of the articular cartilage (Fig. 3, Fig. 5b and c). The Mankin score of these groups was as high as 11.38 ± 1.19 (Fig. 5a). Twelve weeks after surgery, the OA lesions of the DMM joints were further aggravated and Mankin scores increased to 13.00 ± 1.22 (Fig. 5a). These findings suggest that estrogen deficiency accelerates articular cartilage damage. Group B showed similar results on the Sham side, but the cartilage injury of the knee joints on the Sham side showed less severity than those on the DMM side in the same period (Fig. 3, Fig. 5a-c).

At each time point after operation, the cartilage injury of the ipsilateral knee joint in group C was less severe compared with that in group B (Fig. 3, Fig. 5a-c). Eight weeks after operation, moderate OA lesions appeared in the DMM joints of group C; severe loss of local safranin O staining and a small amount of loss in the uncalcified cartilage were also noted. The thickness and area of articular and uncalcified cartilage decreased to varying degrees (Fig. 3, Fig. 5a-c). The Mankin score was 6.06 ± 0.52 and increased to 8.38 ± 0.74 within 12 weeks after the operation. No obvious damage was found in the cartilage of knee joints on the Sham side in the experiment (Fig. 3, Fig. 5a-c).

### Expression of MMP13 in the knee cartilage of mice

**Fig. 6:**
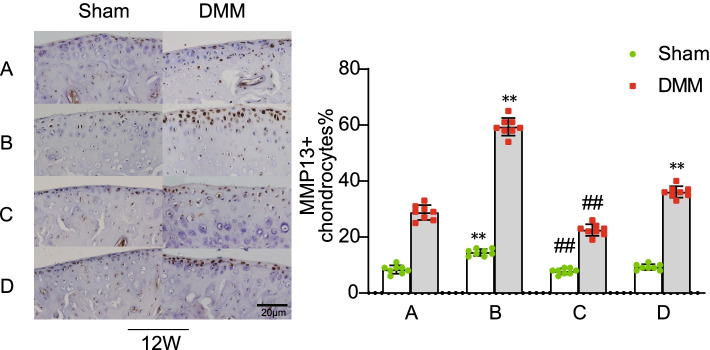
Immunohistochemical expression of MMP13 in the bilateral knee cartilage of mice in groups.(Scale bar = 20 μm). (**A**). female control group; (**B**). ovariectomized group; (**C**). ovariectomized group supplemented with estrogen; and (**D**). male group; Sham, sham operation; DMM, medial meniscus instability operation; Ipsilateral comparison between groups B, D, and A (** *P* < 0.01). Ipsilateral comparison between groups C and B (## *P* < 0.01)

The expression of MMP13 in the knee cartilage of mice in each group was detected by immunohistochemistry 12 weeks after operation (Fig. 6). The expression of MMP13 was mainly concentrated in the uncalcified layer of the cartilage and significantly higher in the knee cartilage on the DMM side than that on the Sham side. The expression of MMP13 in the knee cartilage on the DMM side of group D was higher than that in group A, but no significant difference in MMP13 expression in knee cartilage on the Sham side was found (Fig. 6). Compared with that in group A, the expression of MMP13 in the ipsilateral knee cartilage in group B increased significantly after ovariectomy and the expression of MMP13 in group C decreased significantly after estrogen supplementation.

### Fluorescence staining of apoptotic chondrocytes in mouse knee joints

**Fig. 7:**
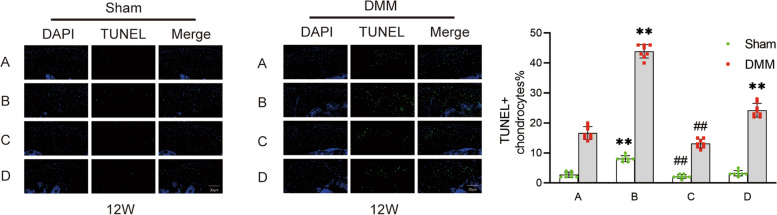
Apoptosis fluorescence staining of chondrocytes in the bilateral knee joints of micein groups.(Scale bar = 30 μm). (**A**). female control group; (**B**). ovariectomized group; (**C**). ovariectomized group supplemented with estrogen; and (**D**). male group; Sham, sham operation; DMM, medial meniscus instability operation; Ipsilateral comparison between groups B, D, and A (** *P* < 0.01). Ipsilateral comparison between groups C and B (## *P* < 0.01)

The apoptosis of knee chondrocytes in each group was measured 12 weeks after surgery. The proportion of apoptotic chondrocytes in the knee joint of mice on the DMM side was higher than that on the Sham side (Fig. 7). The proportion of apoptotic chondrocytes on the DMM side of knee joints in group D was higher than that in group A, but no significant difference in knee cartilage on the Sham side in group D was found. The apoptosis rate of chondrocytes in ipsilateral knee joints in group B was higher than that in group A after ovariectomy (Fig. 7). In addition, the apoptosis rate of chondrocytes in ipsilateral knee joints in group C was lower than that in group B after estrogen supplementation.

### Expression of p-EGFR in the knee cartilage of mice

**Fig. 8:**
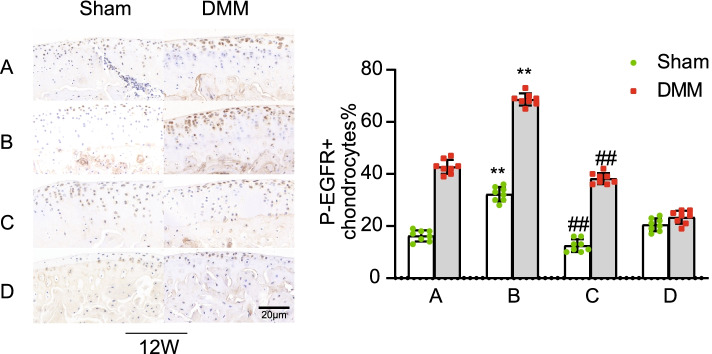
P-EGFR expression in the bilateral knee cartilage of mice in groups.(Scale bar = 20 μm). (**A**). female control group; (**B**). ovariectomized group; (**C**). ovariectomized group supplemented with estrogen; and (**D**). male group; Sham, sham operation; DMM, medial meniscus instability operation; Ipsilateral comparison between groups B and A (* * *P* < 0.01). Ipsilateral comparison between groups C and B (# # *P* < 0.01)

Twelve weeks after surgery, the expression of p-EGFR in the knee cartilage of mice was detected by immunohistochemistry. p-EGFR expression was mainly concentrated in the uncalcified cartilage layer (Fig. 8). The expression of p-EGFR in the knee cartilage on the DMM side was higher than that on the Sham side in female mice. The expression of p-EGFR in the ipsilateral knee cartilage of ovariectomized mice in group B was significantly higher than that in group A (Fig. 8). The expression of p-EGFR in the ipsilateral knee cartilage of mice in group C was significantly lower than that in group B (Fig. 8).

## Discussion

Our results showed that estrogen could delay PTOA progression. Mechanistically, its inhibits the expression of p-EGFR in cartilage on the knee joint surface, and attenuated MMP13 expression and has a protective effect on articular cartilage in female mice.

DMM operation is widely used in studies of animal models of PTOA [19, 20]. In this operation, the medial meniscus-tibial ligament is amputated under a microscope to establish meniscus instability, increase the friction between the meniscus and the bone, and change the stress load of the joint, resulting in PTOA. Glasson et al. [21] found that the pathogenesis of PTOA induced by DMM surgery is slower than that caused by the transection of the anterior cruciate ligament and that the mouse model can well simulate human PTOA. Our results showed no obvious PTOA lesions in Sham knee joints and no damage to the articular cartilage of mice in the female control group. Mankin scores increased slightly and the cartilage thickness decreased slightly in the female control group but revealed no statistical significance. Thus, the differences found were attributed to an increase in age, which leads to a slight degeneration of the articular cartilage. Compared with Sham knee joints in the same period, moderate PTOA lesions, irregularities in the articular cartilage surface, and decreased safranin O staining and cartilage thickness were observed in DMM knee joints as early as eight weeks after operation. Twelve weeks after operation, cartilage lesions in the DMM knee joint were further aggravated. These findings reveal that DMM operation can effectively establish a mouse PTOA model.

Many studies have found [22, 23] that female stress has a protective effect on articular cartilage and can inhibit the progression of OA. The incidence of OA in postmenopausal women is significantly higher than that in men of the same age, thus indicating that estrogen may have an inhibitory effect on the pathogenesis of OA. Sniekers et al. [24] found that a lack of estrogen aggravates articular cartilage injury and susceptibility to OA progression of OA establishing an ovariectomized mouse model. To verify the effect of estrogen on articular cartilage, we established a mouse PTOA model by DMM operation. Our results showed that knee joint cartilage injury on the DMM side of male mice was more serious than that of the female control mice in the same period, but no significant difference in the pathological changes of the cartilage on the Sham operation side was observed, thus indicating that estrogen may have a potential protective effect on articular cartilage. Compared with that of the female control group, the knee cartilage on the Sham operation side of the ovariectomized group showed more serious damage. The DMM side showed similar results, but the degree ofcartilage lesion was significantly more severe. In fact, as early as 8 weeks after operation, severe knee joint PTOA lesions and large areas cartilage defects were found in the uncalcified, and a serious decrease in the area and thickness of articular cartilage. These results suggest that a lack of estrogen accelerates articular cartilage injury. Physiological levels of estrogen can regulate the metabolism of articular cartilage and may have a potential protective effect on articular cartilage and delay the progress of PTOA.

At present, controversy about the effect of estrogen replacement therapy on the protection or repair of OA joint tissue remains, but most experimental results show the beneficial effect of estrogen. Ma et al. [25] found that exogenous estrogen replacement therapy can effectively inhibit articular cartilage injury in mice but has no significant effect on the release of proteoglycans induced by the degradation of the cartilage extracellular matrix. A recent large-scale study of 4766 postmenopausal women in South Korea showed that those who received estrogen replacement therapy have a significantly lower prevalence of symptomatic knee joint OA than those who did not receive estrogen [26]. Our results showed that the knee cartilage injury of female mice was significantly aggravated after ovariectomized, which showed that severe PTOA, supplementation with physiological levels of estrogen could significantly inhibit knee cartilage injury on the DMM and Sham operation sides. These results demonstrate that exogenous estrogen replacement therapy can effectively inhibit articular cartilage injury and delay the progression of PTOA.

Chondrocyte hypertrophy and apoptosis and excessive degradation of cartilage extracellular matrix are important mechanisms in the pathogenesis of OA. Dysregulation of the balance between the catabolism and anabolism of the extracellular matrix is one of the main characteristics of early-stage cartilage injury. MMP13 is one of the most important proteases influencing cartilage extracellular matrix degradation. High MMP13 expression has been observed in the articular cartilage of OA patients [3], and MMP13 gene knockout mice show less cartilage damage in an OA model than ordinary mice [27]. Our study demonstrated that estrogen deficiency can lead to increases in the proportion of apoptotic chondrocytes and MMP13 expression in articular cartilage, whereas estrogen supplementation can significantly decrease chondrocyte apoptosis and MMP13 expression. These results indicate that estrogen can protect the articular cartilage by inhibiting chondrocyte apoptosis and reducing the excessive degradation of the chondrocyte extracellular matrix. Therefore, maintaining the anabolic activity of chondrocytes through estrogen may be an important direction in the treatment of OA.

Although surface chondrocytes play a key role in maintaining articular cartilage and delaying the pathogenesis of OA, whether they are regulated by growth factors and hormones is unknown. The role of the EGFR signaling pathway in regulating cartilage development and extracellular metabolic balance has been gradually recognized. At present, the role of the EGFR signaling pathway in rodent OA models is not clear. It is often affected by OA subtype, OA stage, age, and sex, among other factors, resulting in contradictory results [11, 13]. Huang et al. [28] found that trans-activation of the EGFR signaling pathway in human chondrocytes can enhance the expression of MMP13, which leads to cartilage destruction in joints. Our experiments showed similar results. Specifically, the expression of p-EGFR and MMP13 on the surface of knee cartilage on the DMM side of the mice was significantly higher than that on the Sham-operated side; the articular cartilage on the DMM side was also seriously injured. SHIN and other scholars have shown that inhibition of the EGFR signaling pathway by integrin α1β1 or erlotinib could inhibit articular cartilage injury and delay the progression of PTOA in female mice [12, 13]. Our experimental results showed that estrogen has a negative regulatory effect on the expression of the EGFR signaling pathway in articular cartilage. Specifically, estrogen can inhibit the over-activation and expression of the EGFR signaling pathway and reduce the destruction of articular cartilage. Thus, the hormone alleviates PTOA progression by inhibiting the EGFR signaling pathway in articular cartilage. However, the specific mechanisms of estrogen and the EGFR signal pathway in articular cartilage require further research.

Weight gain is a known risk factor for OA [24]. Coggon et a.l [29] reported that subjects with overweight were 6.8 times more likely to develop knee OA than normal-weight controls. Griffin et al. [30] show that a high-fat diet induces osteoarthritic changes in the knee joint in proportion to fat gain in female mice. Our results showed that estrogen deficiency can lead to weight gain in mice, but a lack of correlation between body weight and the severity of PTOA was observed. This finding may indicate that weight gain is not the main factor aggravating the progression of PTOA in this model. Although DMM surgery has been widely used in studies of animal models of PTOA, it is not formed spontaneously and influenced to some extent by human factors. The factors affecting the experiment will be addressed in a follow-up study.

## Conclusions

This study confirmed the regulatory effect of estrogen on PTOA. Estrogen can protect the articular cartilage by inhibiting the expression of MMP13 and reducing the apoptosis of chondrocytes in female. Our results demonstrate that estrogen can inhibit the activation and expression of the EGFR signaling pathway in articular cartilage in female, but not male mice. And we speculate that estrogen can inhibit the activation of cartilage EGFR signaling pathway by decreasing EGFR transcription in post-traumatic osteoarthritis progression. We will confirmed this speculation in subsequent study.As far as we know, there has been no previous study linking estrogen and EGFR signaling pathway in articular cartilage.The findings provide important clues for the prevention and treatment of PTOA. The specific mechanisms of estrogen and the EGFR signaling pathway in articular cartilage may be intensively studied in future in vitro and clinical experiments.

## Data Availability

The datasets used and/or analyzed during the current study are available from the corresponding author on reasonable request.
